# Neurocognitive function in patients with negative thought disorders

**DOI:** 10.1192/j.eurpsy.2021.2100

**Published:** 2021-08-13

**Authors:** I. Fateev, M. Omelchenko, I. Pluzhnikov

**Affiliations:** 1 Department Of Youth Psychiatry, The Mental Health Research Center, Moscow, Russian Federation; 2 Department Of Adult Neuropsychology And Abnormal Psychology, Moscow Institute of Psychoanalysis, Moscow, Russian Federation

**Keywords:** Schizophrenic spectrum disorder, Neurocognitive function, Executive functions, Negative thought disorders

## Abstract

**Introduction:**

Negative thought disorders are found in various diagnoses in clinical practice. These symptoms may show a possible psychosis continuum and may be taken into account when assessing schizophrenic risk. Neurocognitive functioning of patients with negative thought disorders need to be clarified.

**Objectives:**

Aim of the study is to identify and validate the differences of executive functions between patients with negative thought disorders and patients without thought disorders.

**Methods:**

Used a standardized neuropsychological test battery. There were 15 patients with negative thought disorders (affective disorders, personality disorders, schizophrenic spectrum disorders) and 18 patients with depressive episode without thought disorders in the research. Patients aged 17-25 years. The Mann–Whitney U test and ANOVA were used for statistical analysis.

**Results:**

Significant results were obtained from The Verbal Fluency Test, The Design Fluency Test, The Digit span, The Rey-Osterrieth Complex Figure and Bidstrup’s drawings (All tests have p-values less than 0.05). In the methods listed above, the results in the group of patients with negative thought disorders are significantly lower than in the group of patients without thought disorders.

**Conclusions:**

The data indicate a violation of Executive functions among patients with negative thought disorders: inhibitory control, planning and regulation, working memory, difficulty switching, which related to left frontal lobe dysfunction. A lack of simultaneity and understanding figurative language, which is associated with right hemisphere dysfunction.

**Disclosure:**

The reported study was funded by RFBR, project number 20-013-00772

